# Upregulated MicroRNA-155 Expression in Peripheral Blood Mononuclear Cells and Fibroblast-Like Synoviocytes in Rheumatoid Arthritis

**DOI:** 10.1155/2013/296139

**Published:** 2013-09-17

**Authors:** Li Long, Ping Yu, Yanying Liu, Shiyao Wang, Ru Li, Jinxia Shi, Xiaoping Zhang, Yanmei Li, Xiaolin Sun, Bin Zhou, Liufu Cui, Zhanguo Li

**Affiliations:** ^1^Department of Rheumatology & Immunology, Peking University People's Hospital, 11 Xizhimen South Street, Beijing 100044, China; ^2^Department of Rheumatology & Immunology, Sichuan Academy of Medical Sciences & Sichuan Provincial People's Hospital, Chengdu 610072, China; ^3^Department of Rheumatology & Immunology, Kailuan General Hospital, Tangshan 063000, China; ^4^Department of Rheumatology & Immunology, Weifang Medical University, Weifang 261053, China

## Abstract

*Objective*. This study was to screen for the miRNAs differently expressed in peripheral blood mononuclear cells (PBMC) of RA, to further identify the expression of miR-155 in RA PBMC and fibroblast-like synoviocytes (FLS), and to evaluate the function of miR-155 in RA-FLS. *Methods*. Microarray was used to screen for differentially expressed miRNAs in RA PBMC. miR-155 expression in PBMC and FLS of RA were identified by real-time PCR. Enforced overexpression and downexpression of miR-155 were used to investigate the function of miR-155 in RA-FLS. Expression of IKBKE which was previously identified as the actual target of miR-155 was examined by Western blot and real-time PCR in RA-FLS. *Results*. miR-155 levels were increased in both PBMC and FLS of RA and could be induced by TNF-**α**. Upregulation of miR-155 decreased MMP-3 levels and suppressed proliferation and invasion of RA-FLS. Inverse relationship between the expressions of miR-155 and the MMPs production-related protein IKBKE was found. *Conclusion*. An inflammatory milieu may alter miRNA expression profiles in rheumatoid arthritis. miR-155 is upregulated in RA-FLS, and it may be a protective factor against the inflammatory effect in part by attenuating expression of IKBKE.

## 1. Introduction

Rheumatoid arthritis (RA) is characterized as a systemic autoimmune inflammatory disease which predominantly affects multiple peripheral joints. Accumulating evidence suggests that the participation of inflammation-associated cells as well as the production of proinflammatory mediators by them plays a key role in pathogenesis of RA. Besides, resident fibroblast-like synoviocytes (FLS) contribute significantly to the perpetuation of the disease, and they may even play a role in its initiation. The local production of cytokines and chemokines by these cells accounts for many of the pathologic and clinical manifestations of RA. However, the exact mechanisms are yet unknown.

MicroRNAs (miRNAs) are a large family of highly conserved noncoding genes that regulate gene expression by translational repression or mRNA degradation. To date, hundreds of miRNAs have been identified in the human genome [1, 2], and up to 30% of all protein-encoding genes are estimated to be regulated by them [3]. miRNAs are transcribed in larger precursors and after processing are transported into the cytoplasm. The ~22 nt mature miRNAs control gene expression at the posttranscriptional level by binding to target messenger RNAs (mRNAs) and initiating either their cleavage or a reduction in the translational efficiency [4–7].

Increasing evidence has linked miRNA regulatory activities with human diseases, most notably cancer. More recently, biochemical and genetic studies have begun to reveal the physiological functions of individual miRNAs in immunity. Several articles which support an important role for these tiny RNAs in immune modulation have revealed the role of miRNAs involved in RA pathogenesis [8–11]. To provide more evidence of miRNAs involvement in RA, we compared the levels of miRNAs expression in RA patients with normal controls via microarray profile and then chose miR-155 which had been shown to influence the immune system for further study. We explored the functional role of miR-155 in RA-FLS and the involved mechanisms. Our findings suggest that miR-155 plays a key role in regulating MMP-3 production, as well as the proliferation and invasion of RA FLS.

## 2. Methods

### 2.1. Patients and Controls

Blood samples were obtained from 26 patients admitted to the Department of Rheumatology and Immunology, People's Hospital, Peking University, between October 2007 and January 2008 (24 women, 2 men; median age 56.2 years, range 21–61), who fulfilled the American College of Rheumatology (ACR) criteria for RA. The median disease duration of RA was 9.2 ± 5.2 years. Twenty-three blood donors were included as normal controls.

For RA patients, anti-CCP antibody was tested using the second-generation ELISA kit (Euroimmun, Germany) and values >1.0 were considered positive. Erythrocyte sedimentation rate (ESR) was measured by the Westergren method, and values ≤15 mm/h for males and ≤20 mm/h for females were considered normal. Serum C-reactive protein (CRP) and rheumatoid factor (RF) were examined by an immunonephelometry method. Values >7.9 mg/L for CRP and >20 IU/mL were considered positive for RF.

### 2.2. Isolation and Culture of FLS of RA and OA Patients

Synovial tissues were obtained from patients with RA (*n* = 10, females, ages 30 to 60 years) and osteoarthritis (OA, *n* = 8, females, ages 40 to 70 years) at the time of knee replacement surgery. To isolate synovial fibroblasts, synovial tissue specimens were minced and digested with dispase at 37°C for 60 minutes. After washing, cells were grown in RPMI 1640 (Gibco-Invitrogen, Basel, Switzerland) supplemented with 10% fetal calf serum (FCS) and 50 IU/mL penicillin/streptomycin. Cultures of RA-FLS and OA-FLS were maintained at 37°C in a humidified atmosphere of 5% CO_2_. All synovial fibroblasts between passages 4 and 6 were subjected to experimental procedures. The procedure was approved by the ethical committee of the Peking University People's Hospital. All patients gave written informed consent.

### 2.3. Microarray Experiments

Small RNA was isolated from peripheral blood mononuclear cells (PBMC) of 5 RA patients and 5 normal controls, using the miRNeasy Mini Kit (Qiagen Sciences, MD, USA) according to the manufacturer's protocol, and 0.4 *μ*g/sample of small RNA of the two groups was mixed, respectively. Small RNA mixture of RA and normal controls were labeled Cy3 and Cy5, respectively. Then these mixtures were hybridized to a MiRCURY locked nucleic acid (LNA) microarray (LC Sciences, USA) containing 454 LNA-modified oligonucleotide probes for human, as annotated in the miRBase release 10.1 (http://microrna.sanger.ac.uk/sequences/). All microarray data are based on six probe replicates for each miRNA prediction.

The fluorescence signals were collected and converted to digital signals: the difference between RA and normal group was demonstrated by log2 = log2 (signal Cy3/ signal Cy5). Positive value of log2 means upregulation, and negative value of log2 means downregulation.

### 2.4. Cell Stimulation

FLS from 3 RA patients and PBMC from a separate group of 6 RA patients were transferred to 6-well (2 × 10^5^ cells in 3 mL RPMI 1640 medium with 10% fetal bovine serum/well) cluster plates separately. They were treated with recombinant human TNF-*α* (20 ng/mL, R&D Systems, USA) and then incubated for 48 hours under an atmosphere of 5% CO_2_. Cells were washed twice with cold PBS prior to analysis. All experiments in our study including the following study were performed independently at least three times for each point described.

### 2.5. Quantitative Real-Time PCR (qRT-PCR)

miRNA qRT-PCR was performed using the SYBR Green miRNA assay (Hairpin-it miRNAs Real-Time PCR Quantitation Kit, GenePharma Ltd., China) to detect only the mature form of the miRNA under the following conditions: degeneration at 95°C for 3 min, 40 cycles of 15 s at 95°C, 30 s at 55°C, and 30 s at 72°C. U6 snRNA was used as an endogenous control for data normalization. The 20 *μ*L reaction system contains 2× real-time PCR master mix 10 *μ*L, primers (100 nM) 1 *μ*L, cDNA 2 *μ*L, and Taq DNA polymerase (5 U/*μ*L) 0.2 *μ*L.

IKBKE mRNA qRT-PCR with SYBR Green was performed using an Applied Biosystems 7300 Sequence Detection System in a 20 *μ*L PCR mixture containing 10 *μ*L of 2× real-time PCR master mix, 1.0 *μ*L of primers (100 nM), 0.2 *μ*L of Taq DNA polymerase (5 U/*μ*L), and 2 *μ*L of RT product. The reaction condition was 3 min at 95°C for degeneration and 40 cycles of 15 s at 95°C, 30 s at 55°C, and 30 s at 72°C. The primers are IKBKE forward 5′-TGTCTTCAGCAAACGGCAT-3′, reverse 5′-GGTCGCCAGGTCTCAGG-3′ and GAPDH forward 5′-TGGTATCGTGGAAGGACTCA-3′, reverse5′-GTAGAGGCAGGGATGATGTTC-3′.

The relative miRNA or mRNA expression was calculated using the 2^−ΔΔCt^ method.

### 2.6. Cell Transfection with miRNA Mimic or Inhibitor

RA-FLS were transfected in 12-well plates (5 × 10^4^ cells/well) using Lipofectamine 2000 reagent (Invitrogen) according to the manufacturer's protocol, with 100 nM (final concentration) of synthetic mature miR-155 molecule (miR-155 mimic), antagomir antisense to mature miR-155 (miR-155 inhibitor), or a scrambled control serving as a negative control (NC) (GenePharma Ltd., China), which was carried out 24 hours prior to stimulation with TNF-*α* (20 ng/mL). Twenty-four hours after stimulation, expression levels of the MMPs and TGF-*β* were measured. In separate experiments, RA-FLS were transfected with miR-155 mimic, miR-155 inhibitor, or scrambled control. Forty-eight hours after transfection and twenty-four hours after stimulation, apoptotic status and invasive behavior of RA-FLS were assayed separately.

For proliferation assays, RA-FLS were transfected in 96-well plates (5 × 10^3^ cells/well) with 100 nM (final concentration) of synthetic mature miR-155 molecule (miR-155 mimic), miR-155 Inhibitor, or a scrambled control under the stimulation of TNF-*α* (20 ng/mL). The group in which RA-FLS were cultured alone served as negative controls.

### 2.7. Apoptosis Detection

Apoptosis of RA-FLS was measured after transfection with miR-155 mimic, miR-155 inhibitor, or scrambled control for 48 hours at 37°C. Apoptosis was measured using flow cytometric detection of annexin V binding and propidium iodide (PI) staining (annexin V-FITC) according to the manufacturer's instructions.

### 2.8. Proliferation Assay

RA-FLS were performed in triplicate in 96-well flat-bottom microtitre plates (Corning, NY) in a total volume of 0.2 mL in RPMI 1640 supplemented with 10% FCS. After transfection with miR-155 mimic, miR-155 inhibitor, or scramble control, the RA-FLS were incubated in a humidified atmosphere of 5% CO_2_ at 37°C for 48 h. Eighteen hours before the termination of culture, 1 *μ*Ci of ^3^H-thymidine (GE Healthcare, Amersham, UK) was added to each well. Cells were harvested onto nitrocellulose, and the radioactivity incorporated was counted in a scintillation counter. The FLS proliferation was represented as the incorporated radioactivity in c.p.m, and the results were expressed as c.p.m. ± S.D. of the mean. All experiments in our study including the following study were performed independently at least three times for each point described.

### 2.9. Transwell Culture

FLS were cultivated in the inner chamber of a 6.5 mm diameter Transwell plate with an 0.4 *μ*m pore size membrane (Corning) in RPMI 1640 without FCS, while medium in the lower chamber was RPMI 1640 supplemented with 10% FCS. After 48 h, the invasive behavior of FLS was assayed using the Cytoselect 24-Well Cell Migration and Invasion Assay (Cell Biolabs Inc., San Diego) according to the manufacturer's instructions. Briefly, FLS would be distributed to wells containing FCS. Forty-eight hours later, the inserts were stained with the cell stain solution and the OD 560 nm was measured by a plate reader.

### 2.10. Western Blot Analysis

Equal amounts of protein samples (20 *μ*g) extracted from the cells were separated by SDS-PAGE and transferred onto nitrocellulose membrane. After blotting, membrane was probed with primary antibodies against IKBKE (R&D Systems, USA) and *β*-actin (R&D Systems, USA) and subsequently incubated with secondary antibody. The membrane was then washed and visualized by ECL detection system. In this experiment, *β*-actin expression was shown as protein loading control.

### 2.11. Statistical Analysis

Data were analyzed with SPSS 13.0 for Windows. Results were expressed as mean ± S.D. Statistical analysis was done with Student's *t*-test for comparison of two groups and ANOVA for multiple comparisons. Spearman correlation coefficients were used to assess the correlation between miRNA expression and laboratory data. In all the cases, differences with *P* < 0.05 were considered statistically significant.

## 3. Results

### 3.1. Higher Expression Level of miR-155 in RA PBMC Identified by Microarray Experiments

MicroRNA microchip experiments revealed that RA patients and normal controls show significantly characteristic differences in microRNA expression pattern. Forty-six differently expressed miRNAs were identified (*P* < 0.05, data not shown), and 14 of them were significant in expression level between RA patients and healthy controls (*P* < 0.05, value of log2 >1 or <−1, Table 1). Among these miRNAs, miR-155 significantly increased in PBMC of RA.

### 3.2. Trend of Increased miR-155 Expression in RA PBMC Tested by qRT-PCR

**Figure 1:**
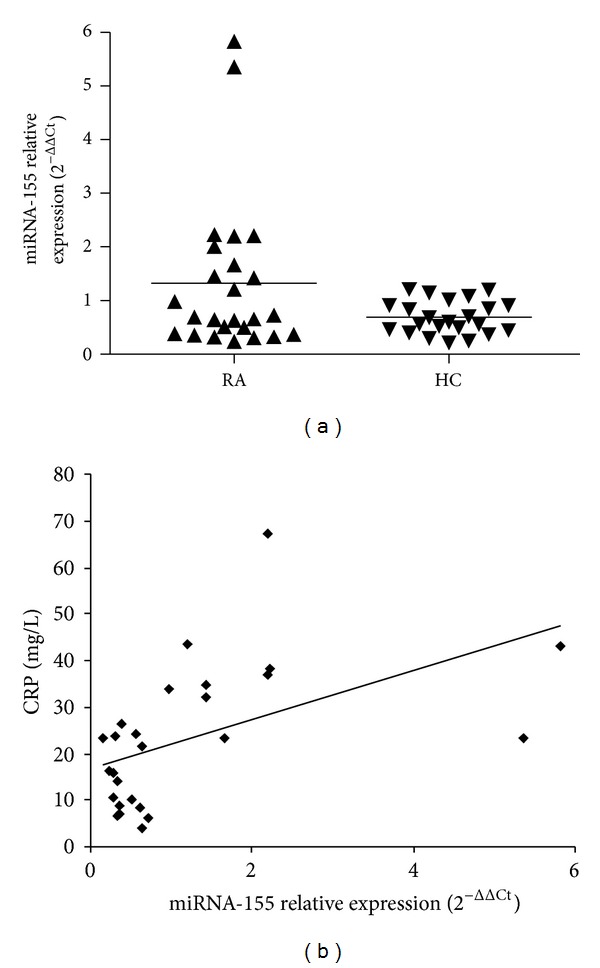
Validation of miR-155 expressions using qRT-PCR and correlation assessment between miR-155 and CRP. (a) Trend of miR-155 overexpression was found in RA PBMC compared to normal controls (1.29 ± 1.42, 0.69 ± 0.31, resp., *P* = 0.053). Triplicate assays were done for each RNA sample and the relative amount of each miRNA was normalized to U6 snRNA. (b) A positive correlation was found between miR-155 expression in PBMC and CRP level of RA patients (*r* = 0.56, *P* < 0.05). RA: rheumatoid arthritis, HC: healthy controls.

Recent studies have shown that miRNA-155 was involved in RA inflammation [7–9], and our microarray results also showed increased miR-155 expression in PBMC of RA. Based on these data, expression of miRNA-155 in RA PBMC was chosen for further identification by qRT-PCR. Increased miR-155 expression was observed in RA PBMC compared with normal controls (*n* = 26, 23, resp.), though the difference between them did not reach statistical significance (*P* = 0.053, Figure 1(a)).

### 3.3. Association between miR-155 and Laboratory Features in RA

To determine the effect of miR-155 expression in RA, the associations between miR-155 and laboratory features in RA patients were analyzed. A positive correlation was found between miR-155 and serum CRP level (*r* = 0.56, *P* < 0.05, Figure 1(b)). However, there is no correlation between miR-155 and other laboratory features such as RF, anti-CCP, and ESR.

### 3.4. Upregulated Expression of miR-155 by Stimulation of TNF-*α* in RA PBMC

**Figure 2:**
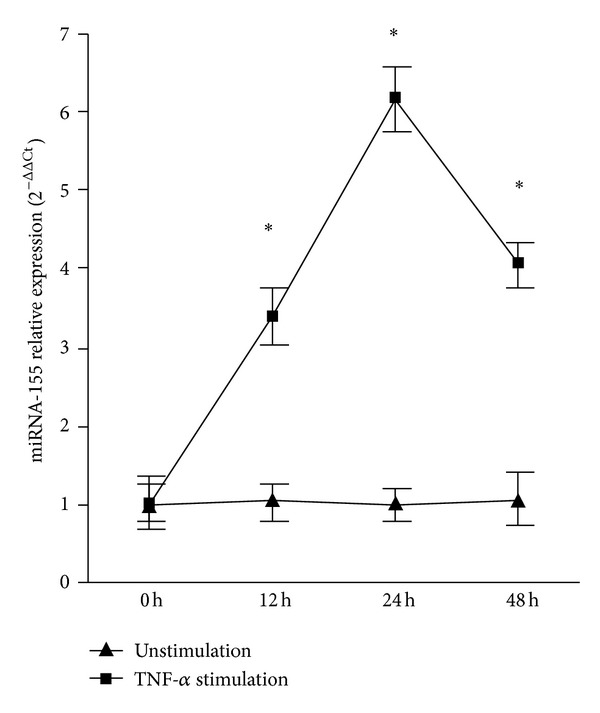
Induction of miR-155 expression in RA PBMC by TNF-*α*. The upregulation of miR-155 upon stimulation of RA PBMC, as compared with that in unstimulated control cultures (*n* = 6), was about 3.31-fold with TNF-*α* stimulation after 12 hours, and 6.12-fold after 24 hours, and 3.79-fold after 48 hours. *P < 0.05.

To evaluate the stimulatory effect of proinflammatory mediators on miR-155, we stimulated RA PBMC with TNF-*α*, which were known to be critically involved in the development of inflammation and destruction of RA. Expression of miR-155 was significantly upregulated in RA PBMC after TNF-*α* stimulation. Such enhanced miR-155 expression was observed after 12 hours when cultured with stimulant, peaking at 24 hours and decreasing at 48 h (Figure 2).

### 3.5. Increased miR-155 Expression and Trend of Upregulation by TNF-*α* on RA-FLS

**Figure 3:**
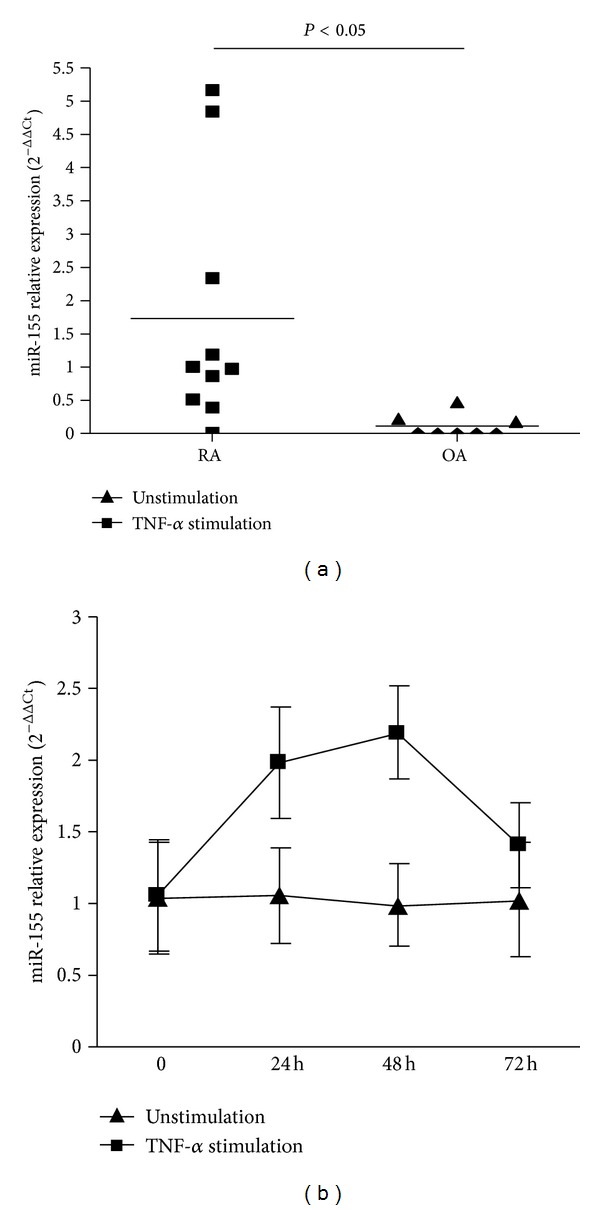
Constitutive expression and inductive expression of miR-155 in RA-FLS. (a) Overexpression of miR-155 in cultured RA-FLS (*n* = 10) compared to OA-FLS (*n* = 8, *P* < 0.05, 1.79 ± 1.94, 0.11 ± 0.17, resp.). (b) Trend of up-regulation by TNF-*α* on RA-FLS (*n* = 3). The up-regulation of miR-155 upon stimulation of RA-FLS, as compared with that in unstimulated control cultures, was about 1.87-fold with TNF-*α* stimulation, after 24 hours (*P* = 0.068); 2.22-fold after 48 hours (*P* = 0.060); and 1.38-fold after 72 hours (*P* = 0.080).

Since RA-FLS contribute significantly to the pathogenesis of RA, we assessed the expression of miR-155 in cultured RA-FLS (*n* = 10) and found 16.27-fold of overexpression compared with OA-FLS (*n* = 8, *P* < 0.05, Figure 3(a)). Considering that TNF-*α* is the most important cytokine that essentially triggers inflammation and joint destruction in RA synovium, we next investigated the regulation of miR-155 by TNF-*α*. Although no statistical significance was reached, we observed an obvious trend of upregulation of miR-155 after treatment of RA-FLS with TNF-*α*. Such increased expression was found when stimulated for 24 h, appearing to peak at 48 h and to decrease slightly at 72 h (Figure 3(b)).

### 3.6. Effect of miR-155 on the Secretion of MMP-3, MMP-9, and TGF-*β* from RA-FLS

**Figure 4:**
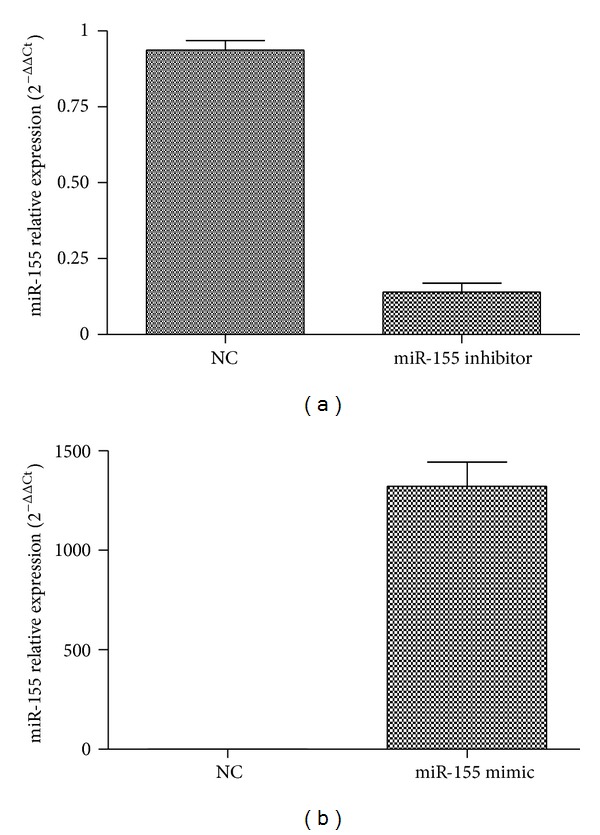
Altered expression of miR-155 by transfection of miR-155 inhibitor and mimic separately. (a) Transfection of miR-155 inhibitor (100 nM) for 48 h significantly decreased miR-155 level by at least 80% in RA-FLS. (b) Transfection of miR-155 mimic (100 nM) for 48 h induced at least 1000-fold increase in miR-155. miR-155 level was determined using qRT-PCR. Three independent experiments were carried out, and each experiment was done in three replicates.

**Figure 5:**
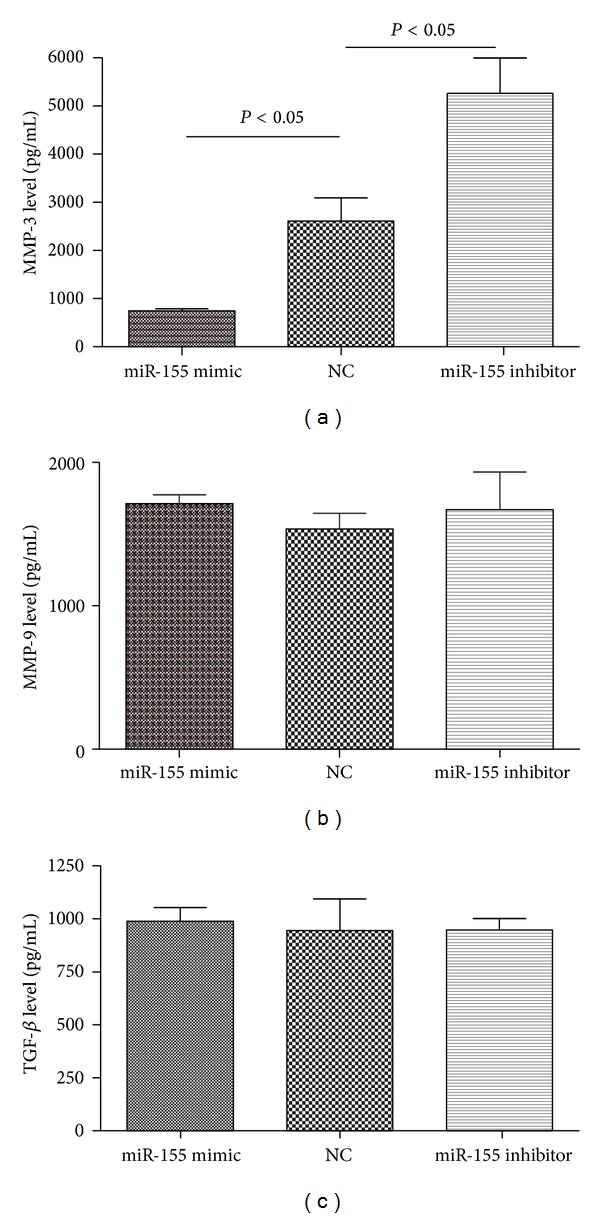
Effect of miR-155 on cytokines secretion of RA-FLS. (a) Increased level of miR-155 suppressed MMP-3 expression (*n* = 3, *P* < 0.05, 747.61 ± 72.19), while decreased level of miR-155 enhanced MMP-3 expression compared to NC in RA-FLS (*n* = 3, *P* < 0.05, 5263.57 ± 1260.16, 2605.23 ± 843.58, resp.). (b) Secretion of MMP-9 was not affected whether miR-155 expression was increased or decreased in RA-FLS compared to NC (*n* = 3, *P* > 0.05, 1712.28 ± 106.41, 1671.08 ± 452.25, 1535.36 ± 192.23, resp.). (c) Secretion of TGF-*β* was not affected whether with increased or decreased expression of miR-155 in RA-FLS compared to NC (*n* = 3, *P* > 0.05, 988.22 ± 113.55, 945.88 ± 94.56, 943.86 ± 258.72, resp.). The cells were transfected with 100 nM miR-155 mimic or miR-155 inhibitor for 48 h under 20 ng/mL TNF-*α* stimulation. Each experiment was in three replicates.

To measure the effect of miR-155 on the cytokine secretion of RA-FLS, we transfected RA-FLS with miR-155 mimic and miR-155 inhibitor separately, which are known to increase/decrease the cellular levels of mature miR-155. Following SYBR Green, qRT-PCR was carried out to confirm the effective upregulation/downregulation of miR-155 in RA-FLS on the level of mature miR-155 (Figure 4). Expression of MMP-3, MMP-9, and TGF-*β* as markers of the destructive and inflammatory properties of RA-FLS was detected. With up-regulated miR-155, MMP-3 levels decreased by 71.3%, while MMP-9 and TGF-*β* levels were not significantly changed (Figure 5). When the endogenous expression of miR-155 in RA-FLS was silenced, we found that MMP-3 levels were significantly enhanced, while MMP-9 and TGF-*β* levels were not influenced (Figure 5).

### 3.7. Suppression of RA-FLS Proliferation by miR-155

**Figure 6:**
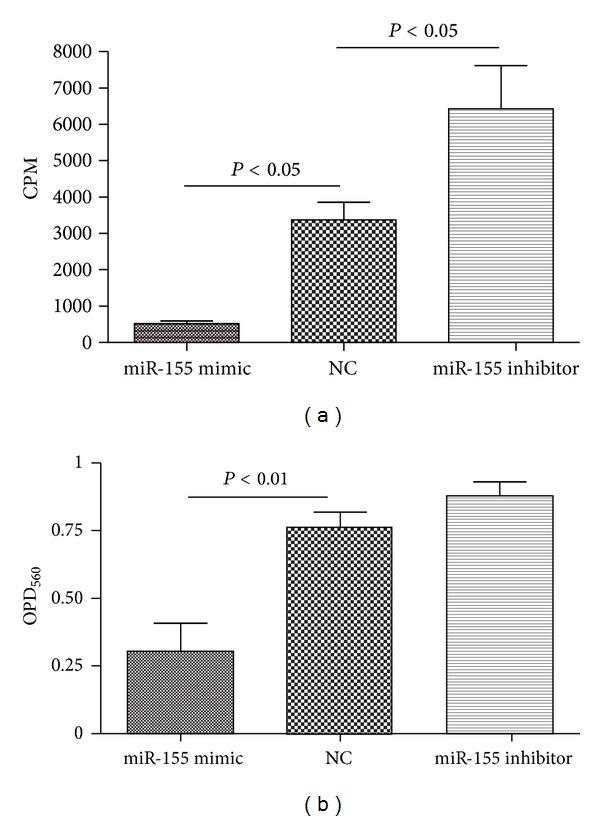
Suppression of RA-FLS proliferation and invasion by miR-155. (a) Cell proliferation was evaluated using ^3^H-thymidine incorporation assay. Increased level of miR-155 reduced proliferation of RA-FLS, while decreased level of miR-155 enhanced proliferation of RA-FLS compared to NC (*n* = 5, *P* < 0.05, 525.53 ± 161.21, 6424.70 ± 2659.66, 3368.60 ± 1084.50, resp.). (b) Transwell invasive assay was used to assess the effect of miR-155 on invasion of RA-FLS. Transfection of miR-155 mimic reduced RA-FLS invasion significantly compared to NC (*n* = 3, *P* < 0.01, 0.31 ± 0.18, 0.76 ± 0.10, resp.), while transfection of miR-155 inhibitor only showed the trend of increased invasion compared to NC (*n* = 3, *P* > 0.05, 0.88 ± 0.09, 0.76 ± 0.10, resp.). Each experiment was done in three replicates.

To assess the possible role of miR-155 in controlling proliferation, we transfected miR-155 mimic or miR-155 inhibitor into RA-FLS. Cell proliferation and viability were determined using the ^3^H-thymidine incorporation assay. As shown in Figure 6(a), reducing the endogenous miR-155 brings about enhancement of RA-FLS proliferation by about 1.91-fold, and inhibition of cell proliferation to about 84.40% of control level was found when miR-155 was up-regulated.

### 3.8. Suppression of RA-FLS Invasion by miR-155

The invasive behavior of RAFSs was assayed using the Cytoselect 24-Well Cell Migration and Invasion Assay. RA-FLS showed a diminished invasion when transfected with miR-155 mimic. Trend of enhanced invasion was observed when endogenous expression of miR-155 was silenced (*P* > 0.05, Figure 6(b)).

### 3.9. No Effect of miR-155 on RA-FLS Apoptosis

**Figure 7:**
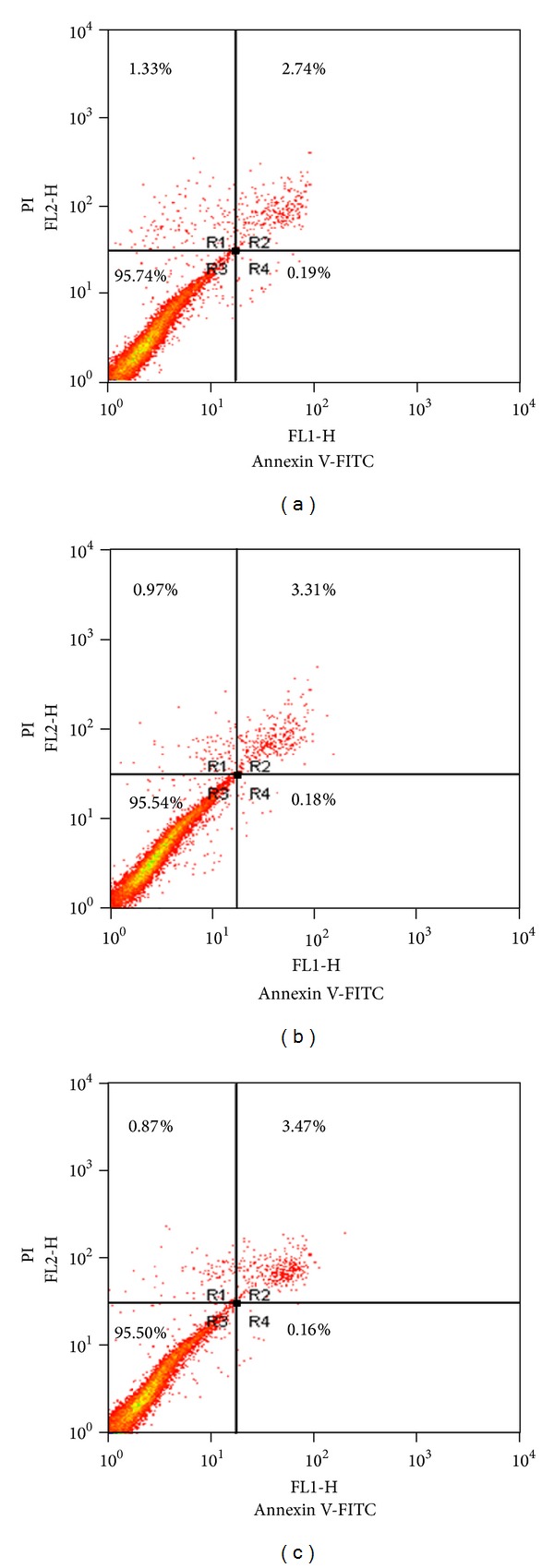
Effect of miR-155 on RA-FLS apoptosis. (a) Effect of miR-155 mimic on RA-FLS apoptosis. The percentage of apoptotic cells (annexin V-positive/PI-negative) was 0.20 ± 0.03%. (b) Effect of miR-155 inhibitor on RA-FLS apoptosis. The percentage of apoptotic cells was 0.20 ± 0.02%. (c) Effect of scrambled control on RA-FLS apoptosis. The percentage of apoptotic cells was 0.17 ± 0.02%. Representative results are shown. There was no statistical significance among these groups (*n* = 4, *P* > 0.05).

To test whether or not the inhibitory effect of miR-155 on RA-FLS proliferation was associated with RA-FLS apoptosis, the percentage of apoptotic cells was evaluated by using the annexin V/PI staining. The percentage of PI and annexin V+ cells after transfection of miR-155 mimic was 0.20 ± 0.03%, which was similar to that when transfected with miR-155 inhibitor or NC control (*P* > 0.05, Figure 7).

### 3.10. Identification of IKBKE as Direct Targets of miR-155 in RA-FLS

**Figure 8:**
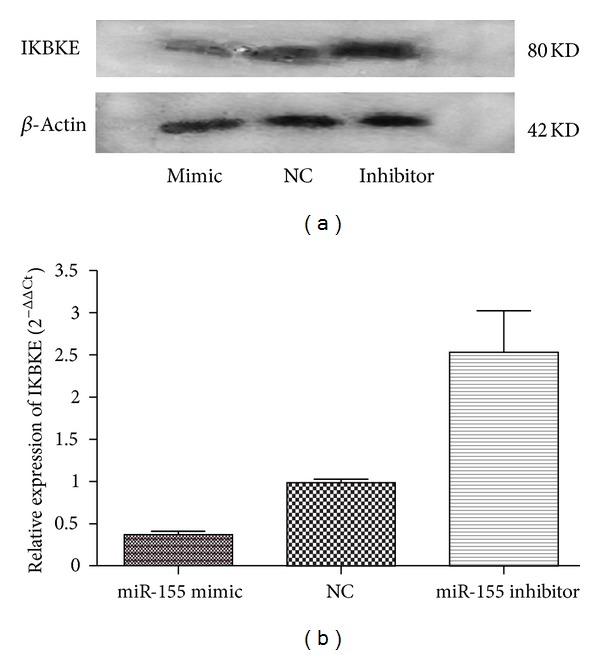
Regulation of miR-155 on IKBKE expression in RA-FLS. (a) Western blot analysis of IKBKE protein expression in miR-155 mimic and miR-155 inhibitor-treated RA-FLS. A representative result of 3 independent experiments is shown. Increased level of miR-155 reduced expression of IKBKE protein, while decreased level of miR-155 enhanced expression of IKBKE protein. *β*-Actin was used as an internal control. (b) Quantitative real-time PCR identified IKBKE mRNA levels in miR-155 mimic and miR-155 inhibitor-treated RA-FLS. Trend of reduced expression of IKBKE mRNA by increased level of miR-155 and enhanced expression of IKBKE mRNA by decreased level of miR-155 compared to NC (*n* = 5, *P* > 0.05, 0.37 ± 0.09, 2.53 ± 1.10, 0.99 ± 0.09, resp.) was observed.

IKBKE transcripts were previously identified as actual targets of miR-155 using a luciferase assay [12]. IKBKE was recently reported to play a key role in MMP gene expression and subsequent joint destruction in arthritis [13], so we investigated whether IKBKE was a target mRNA of miR-155 in RA-FLS. The effects of miR-155 overexpression/downexpression on IKBKE protein expression level were determined by Western blot. We found that miR-155 over-expression decreased IKBKE protein level compared to miRNA negative controls (Figure 8(a)). Additionally, the miR-155 inhibitor increased IKBKE protein level (Figure 8(a)). Most miRNAs function as inhibitors of target protein translation, but in a few cases, they can also induce mRNA cleavage. Therefore, we additionally determined the effects of miR-155 on IKBKE mRNA level by qRT-PCR. In comparison with the NC control, there was a significant trend of decrease in IKBKE mRNA levels in cells transfected with miR-155 and increase in IKBKE mRNA level when miR-155 was inhibited (Figure 8(b)), although no statistical significance was reached. These results showed that miR-155 down-regulated IKBKE mainly at protein level.

## 4. Discussion

Despite an increased understanding of the posttranscriptional regulation of gene expression via miRNA-mediated RNA interference (RNAi), the importance of miRNAs in the immune system development and response has only recently become evident. Several miRNAs involved in innate and/or adaptive immunity have been identified. Of these, evidence of miR-155 function in normal immune response has been reported.

Recent reports have shown that miR-155 is required for normal immune function. miR-155-null mice have fewer class-switched antibodies after immunization due to a failure to select high affinity plasma B cells [14, 15], indicating that miR-155 is required for B cell response. As with B cells, it seems that miR-155 is involved in T cell differentiation. miR-155-null mice showed reduced IL-2 and IFN-*γ* production [16], as well as an increased propensity of T cells to differentiate into Th2 rather than Th1 cells. References [14, 16] suggest miR-155 is critical in T cell differentiation.

Considering the involvement of miR-155 in immune response, studies focused on its role in autoimmunity diseases, such as RA, have emerged recently. Stanczyk et al. reported increased miR-155 expression in RA synovial fibroblasts compared to osteoarthritis synovial fibroblasts, which is the first study about the involvement of miR-155 in RA [9]. Pauley et al. observed that RA PBMC exhibited increased miR-155 compared to normal controls [8]. Here we investigated the expression of miR-155 in larger populations and showed a 1.9-fold increase in the average relative expression of miR-155 in RA PBMC compared to normal controls and 16.27-fold increase in RA-FLS compared to OA-FLS, which is similar to published reports. These findings indicate that the constitutive expression of miR-155 is increased in RA inflammatory cells.

Since TNF-*α* is an important proinflammatory cytokine shown to be a causative factor in RA and highly elevated in both serum and synovial fluid from patients with RA, we analyzed the expression levels of miR-155 in RA PBMC and RA-FLS upon stimulation with TNF-*α*. Both of them showed significantly up-regulated miRNA-155 expression upon TNF-*α* stimulation. These results suggested that increased level of miR-155 may be correlated with the abundant inflammatory cytokines of RA.

Our study also showed a positive correlation between miR-155 and CRP in RA patients. As CRP level is associated with high RA activity, it is reasonable to speculate that the expression of miR-155 is a potential marker indicating RA disease activity. Further studies involving a larger patient cohort are needed to fully determine whether miRNA expression can serve as a marker for disease activity; also required is an evaluation of the correlation between miRNA expression level and parameters reflecting disease activity, such as DAS28. Dynamic observations of miRNA expression are also needed to further validate the value of miRNA in disease activity assessment.

To further investigate the possible role of miR-155 in RA pathophysiology, we analyzed the effect of miR-155 on RA-FLS function. RA-FLS are now considered as active players in the complex intercellular network of RA, by producing a variety of cytokines/angiogenic factors and matrix degrading enzymes as well as having aggressive and invasive behaviours. We found that silencing expression of miR-155 can significantly promote MMP-3 production and enhance proliferation of RA-FLS, which suggested that endogenous expression of miR-155 plays a key role in regulation of MMP-3 production and proliferation of RA-FLS. When transfected with miR-155 mimic, RA-FLS not only presented less MMP-3 secretion and proliferation, but also showed less aggressive behavior. Such findings revealed a possible therapeutic significance for miR-155. In addition, annexin V/PI staining showed that miR-155 induced no significant apoptosis of RA-FLS, which suggested that the effect of miR-155 on RA-FLS proliferation had no association with cell apoptosis.

Recently Stanczyk et al. reported that enforced expression of miR-155 in RA-FLS repressed the levels of MMP-3 [9], which is in line with our study. They raised the possibility of an indirect action of miR-155 on the control of expression of MMP-3 by synergistic down-regulation of several signal-transducing proteins because MMP-3 has not been predicted (by miRGen analysis) to be a direct target of miR-155. In our study, we further investigated the possible mechanism involved. We did not find that MMP-3 transcript was a direct target of miR-155, using computational prediction provided by miRBase online searching program. However, we noticed that among reported, experimentally validated direct targets of miR-155 by luciferase report assay, after transfection with the relevant 3′UTR portion of mRNA [17], IKBKE (IKK*ε*) was confirmed to directly increase the cytokine-mediated production of MMP-3 and MMP-13 by RA-FLS through phosphorylating c-Jun [13]. It prompted us to explore whether miR-155 regulated MMP-3 expression via targeting IKBKE in RA-FLS. We identified that protein levels of IKBKE were suppressed/enhanced when miR-155 was overexpressed/downregulated in RA-FLS. These results strongly suggested that IKBKE was the direct target of miR-155 in RA-FLS. It was tempting to speculate that miR-155 at least in part suppressed the cytokines-mediated production of MMP-3 through the down-regulation of IKBKE in RA-FLS.

Besides attenuating secretion of MMP-3, miR-155 was also shown to have potent antiproliferative and anti-invasive effects in RA-FLS. To date, there is no evidence showing that miR-155 directly targets key regulators of cellular proliferation and invasion, such as c-myc, ERK, and P38. MMPs especially MMP-3 were reported to be associated with proliferation and invasion of RA-FLS [18]. Since miR-155 can downregulate production of MMP-3 on RA-FLS, it is reasonable to speculate that decreased MMP-3 induced by miR-155 at least in part contributes to the suppression of proliferation and invasion.

In summary, our observation of increased miR-155 expression in both RA PBMC and RA-FLS, as well as significant up-regulation of miR-155 induced by TNF-*α*, suggests that such an inflammatory mediator which is usually elevated in serum and synovial fluid may at least in part contribute to increased expression of miR-155 in RA. Further functional analyses have shown that endogenous miR-155 decreases MMP-3 production and attenuates proliferation of RA-FLS in vitro, which may be a protective factor against the inflammatory effect. Additional experiments have shown that overexpression of miR-155 also inhibits RA-FLS invasion in vitro. These findings raise the possibility of a therapeutic potential of an miRNA-based approach for treating RA.

## Figures and Tables

**Table 1:** List of significantly changed miRNAs of PBMC in RA identified by miRNA microarray.

Probe	Average signal intensity of HC group	Average signal intensity of RA group	log2 (RA group/HC group)*
hsa-miR-29b	24.26	174.27	3.16
hsa-miR-374b	28.45	108.92	1.91
hsa-miR-155	848.69	1,508.07	1.78
hsa-miR-574-5p	613.87	1,688.69	1.43
hsa-miR-483-5p	211.94	525.55	1.24
hsa-miR-625	146.11	284.96	1.09
hsa-miR-149	820.87	1,876.82	1.09
hsa-miR-765	101.13	202.72	1.03
hsa-miR-612	101.76	207.68	1.03
hsa-miR-181c	561.97	140.56	−2.00
hsa-miR-148b	511.93	143.95	−1.75
hsa-miR-181a	5,411.03	2,394.80	−1.19
hsa-miR-146a	1,871.37	863.76	−1.12
hsa-miR-221	3,852.63	1,858.00	−1.08

RA: rheumatoid arthritis, HC: healthy controls; log2 (RA group/HC group) >1 or <−1 indicated the difference was significant; *P < 0.05.
